# Lycopene Inhibits Urotensin-II-Induced Cardiomyocyte Hypertrophy in Neonatal Rat Cardiomyocytes

**DOI:** 10.1155/2014/724670

**Published:** 2014-05-25

**Authors:** Hung-Hsing Chao, Li-Chin Sung, Cheng-Hsien Chen, Ju-Chi Liu, Jin-Jer Chen, Tzu-Hurng Cheng

**Affiliations:** ^1^Shin Kong Wu Ho-Su Memorial Hospital, Taipei 111, Taiwan; ^2^Department of Surgery, School of Medicine, Taipei Medical University, Taipei 110, Taiwan; ^3^Department of Biological Science and Technology, College of Life Sciences, China Medical University, Taichung 40402, Taiwan; ^4^Division of Cardiology, Department of Internal Medicine, Shuang Ho Hospital, Taipei Medical University, New Taipei City 23561, Taiwan; ^5^Department of Internal Medicine, School of Medicine, College of Medicine, Taipei Medical University, Taipei 110, Taiwan; ^6^Division of Cardiology, Department of Internal Medicine and Graduate Institute of Clinical Medical Science, China Medical University, Taichung 40402, Taiwan; ^7^Institute of Biomedical Sciences, Academia Sinica, Taipei 115, Taiwan; ^8^Department of Biochemistry, School of Medicine, China Medical University, Taichung 40402, Taiwan

## Abstract

This study investigated how lycopene affected urotensin-II- (U-II-) induced cardiomyocyte hypertrophy and the possible implicated mechanisms. Neonatal rat cardiomyocytes were exposed to U-II (1 nM) either exclusively or following 6 h of lycopene pretreatment (1–10 **μ**M). The lycopene (3–10 **μ**M) pretreatment significantly inhibited the U-II-induced cardiomyocyte hypertrophy, decreased the production of U-II-induced reactive oxygen species (ROS), and reduced the level of NAD(P)H oxidase-4 expression. Lycopene further inhibited the U-II-induced phosphorylation of the redox-sensitive extracellular signal-regulated kinases. Moreover, lycopene treatment prevented the increase in the phosphorylation of serine-threonine kinase Akt and glycogen synthase kinase-3beta (GSK-3**β**) caused by U-II without affecting the protein levels of the phosphatase and tensin homolog deleted on chromosome 10 (PTEN). However, lycopene increased the PTEN activity level, suggesting that lycopene prevents ROS-induced PTEN inactivation. These findings imply that lycopene yields antihypertrophic effects that can prevent the activation of the Akt/GSK-3**β** hypertrophic pathway by modulating PTEN inactivation through U-II treatment. Thus, the data indicate that lycopene prevented U-II-induced cardiomyocyte hypertrophy through a mechanism involving the inhibition of redox signaling. These findings provide novel data regarding the molecular mechanisms by which lycopene regulates cardiomyocyte hypertrophy.

## 1. Introduction

It is believed that initial hypertrophic response is beneficial; however, sustained hypertrophy often leads to heart failure, which is the primary cause of mortality and morbidity worldwide and is characterized by progressive deterioration in cardiac function [1]. Maladaptive hypertrophy is triggered by neurohormonal mediators and biomechanical stress [2]. The signaling mechanisms leading to cardiac hypertrophy have been extensively investigated throughout the past decade. Urotensin-II (U-II) is a cyclic peptide that exhibits potent vasoconstriction effects [3]. U-II was identified as being highly expressed in cardiac tissues at sites demonstrating an abundant expression of U-II receptors [3]. In the field of cardiovascular disease (CVD), considerable interest is directed toward U-II because of increasing evidence of its role in the development of cardiac remodeling and dysfunction [4]. U-II is upregulated in the failing heart and promotes cardiomyocyte hypertrophy, in particular through mitogen-activated protein kinases (MAPKs) [5]. Another primary effect of U-II is the increased expression of NAD(P)H oxidase, which is a main source of reactive oxygen species (ROS) [5, 6]. ROS have been reported to play a role in the early initiation of cardiomyocyte hypertrophy [5, 7]. We recently demonstrated that the generation of ROS is involved in U-II-induced hypertrophy, the tyrosine phosphorylation of epidermal growth factor receptors (EGFR), and extracellular signal-regulated kinase (ERK) phosphorylation in rat cardiomyocytes [5]. Our study revealed a mechanism through which ROS can regulate cellular processes [5]. This mechanism involves the transient inhibition of protein tyrosine phosphatases (PTPs) through reversible oxidation of their catalytic cysteine residue, suppressing the dephosphorylation of downstream proteins [5]. Several PTPs regulate the receptor tyrosine kinases associated with various signaling pathways, including EGFR. This reversible oxidation mechanism might explain the link between EGFR transactivation and ROS generation in the U-II signaling pathway. One study reported that the U-II induction of adult cardiomyocyte hypertrophy involves the Akt/glycogen synthase kinase-3beta (GSK-3*β*) signaling pathway [8]. GSK-3*β* was the first negative regulator of cardiac hypertrophy to be identified [9]. Akt, a serine-threonine kinase, has been well characterized as an antiapoptotic kinase and directly inactivates endogenous GSK-3*β* via Ser9 phosphorylation [9]. After dephosphorylating the 3′ position of phosphatidylinositol 3,4,5-triphosphate (PIP3), the phosphatase and tensin homolog deleted on chromosome 10 (PTEN) negatively regulates the phosphatidylinositol 3-kinase (PI3K)/Akt pathway [10]. Furthermore, the cardiac-specific inactivation of PTEN leads to cardiac hypertrophy [10]. PTEN is inactivated by oxidative stress, leading to Akt activation [11]. GSK-3*β* mediates antihypertrophic effects through multiple mechanisms [9]; therefore, GSK-3*β* deactivation during cardiac hypertrophy might represent a potential mechanism for modulating the hypertrophic activity of cardiomyocytes.

Lycopene, a carotenoid compound, is known for its health-promoting ability [12] and strong ability to scavenge free radicals [13–15]. Because of its strong antioxidant properties, lycopene demonstrates the ability to reduce the risk of various chronic conditions such as CVD, coronary heart disease, and atherosclerosis [13]. Furthermore, high plasma lycopene concentrations are associated with the decreased risk of CVD incidence [16]. Thus, lycopene treatment might represent a new therapeutic strategy in treating ROS-related pathophysiological damage. Nevertheless, little is known regarding the effects of lycopene in cardioprotection and the underlying mechanisms during cardiomyocyte hypertrophy. ROS have been shown to play a key role in cardiomyocyte hypertrophy [5, 17]. Therefore, this study was conducted to ascertain how lycopene affects U-II-induced cardiomyocyte hypertrophy and to assess the redox signaling pathway involved in these effects. We determined that lycopene might prevent U-II-induced cardiomyocyte hypertrophy in part by inhibiting the Akt/GSK-3*β* pathway and reducing PTEN oxidation.

## 2. Materials and Methods

### 2.1. Materials

Dulbecco's modified Eagle's medium, fetal calf serum, and tissue culture reagents were obtained from Invitrogen (Carlsbad, CA, USA). The human U-II, lycopene, and all other chemicals of reagent grade were obtained from Sigma-Aldrich (St. Louis, MO, USA). The antibodies used in this study were purchased from New England Biolabs (Ipswich, MA, USA), Santa Cruz Biotechnology (Santa Cruz, CA, USA), and Lab Frontier Co. Ltd. (Seoul, Republic of Korea) (anti-GAPDH).

### 2.2. Cardiomyocyte Cell Culture and Immunofluorescence Microscopy

Primary cultures of neonatal rat ventricular myocytes were prepared and plated at high density (1250 cells/cm^2^) as previously described [5]. The principles of laboratory animal care (Institute of Laboratory Animal Resources, 1996) were followed. Microscopic investigation indicated that the primary myocyte cell cultures contained less than 5% noncardiomyocytes. Before treatment, serum-containing medium was removed from the myocyte cultures and replaced with serum-free medium. To visualize changes in cell size [5], the myocytes were plated on fibronectin-coated coverslips at a density of 5 × 10^5^ cells in 35 mm dishes. After the treatment, the cells were fixed and visualized using mouse anti-*α*-actinin (Sigma-Aldrich) and rhodamine-conjugated anti-mouse antibodies. To reveal the cell nuclei, the same slides were stained with 4′,6-diamidino-2-phenylindole (DAPI; 1 *μ*g/mL) in phosphate-buffered saline (PBS) and 0.5% 1,4-diazabicyclo[2,2,2]octane. Immunofluorescence images were captured using a fluorescence microscope (Eclipse; Nikon, Tokyo, Japan) equipped with a digital camera (DXM1200; Nikon). The cell surface areas were measured by conducting a morphometric analysis of the *α*-actinin-stained cardiomyocytes using NIH Image software (http://rsb.info.nih.gov/nih-image/). The cell size was quantified by measuring the cell surface areas of randomly chosen cells from distinct dishes.

### 2.3. Flow Cytometric Assay of 2′,7′-Dichlorodihydrofluorescein Oxidation

The determination of intracellular ROS production was based on the oxidation of 2′,7′-dichlorodihydrofluorescein (DCFH) to fluorescent 2′,7′-dichlorofluorescein (DCF), as previously described [5]. DCFH was added at a final concentration of 10 *μ*M and incubated for 30 min at 37°C. The cells were then washed once with PBS and maintained in a 1 mL culture medium. After drug treatment, the medium was aspirated and the cells were washed twice with PBS and then dissociated using trypsin. The level of cellular fluorescence was determined using flow cytometry (FACScan; BD Biosciences). The cells were excited using an argon laser at 488 nm, and measurements were taken from 510 to 540 nm.

### 2.4. Western Blot Analysis

The rabbit polyclonal antiphospho-ERK antibody was purchased from New England Biolabs. The anti-ERK, anti-NAD(P)H oxidase-4 (NOX4), and anti-PTEN antibodies were purchased from Santa Cruz Biotechnology Inc. Whole-cell extracts were obtained using a radioimmunoprecipitation assay buffer (10 mM Tris, pH 7.5, 150 mM NaCl, 0.1% SDS, 1.0% Triton X-100, 1% sodium deoxycholate, 5 mM EDTA, 1 mM sodium orthovanadate, 1 mM phenylmethylsulfonyl fluoride, and a complete protease inhibitor cocktail (Roche Diagnostics GmbH, Mannheim, Germany)). The extracts or proteins were separated using SDS polyacrylamide gel electrophoresis, electrotransferred to polyvinylidene difluoride membranes, and probed with antisera followed by horseradish peroxidase-conjugated secondary antibodies. The proteins were visualized using chemiluminescence based on the manufacturer instructions (Pierce Biotechnology Inc., IL, USA).

### 2.5. Protein Synthesis Measurement

To measure the synthesis of the new proteins, the cardiomyocytes were incubated with 1.0 *μ*Ci/mL of [^3^H]-leucine in serum-free medium [5]. The cells were harvested using incubation at 4°C with trichloroacetic acid (5%) followed by solubilization in 0.1 N of NaOH. The radioactivity level was determined using scintillation counting.

### 2.6. Immunoprecipitation and Phosphatase and Tensin Homolog Activity Assay

The cells were lysed at 4°C in lysis buffer (50 mM Tris, pH 7.5, 1% Nonidet P-40, 0.5% sodium deoxycholate, 150 mM NaCl, and protease inhibitors). The PTEN was collected using immunoprecipitation kits (Roche Molecular Biochemicals, Mannheim, Germany) to assess specific antibodies and protein-G-agarose, following the manufacturer instructions [18]. The precipitates were washed with lysate buffer and the PTEN activity level was analyzed using a PTEN Malachite Green Assay Kit (Upstate, Lake Placid, NY, USA). The absorbance was detected at 600 nm. The released phosphate was determined relative to a standard curve.

### 2.7. Statistical Analysis

The data are presented as mean ± SEM. Based on the context, the statistical analysis was performed using a Student's *t*-test or ANOVA, followed by a Dunnett multiple comparison test, using Prism version 3.00 for Windows (GraphPad Software; San Diego, CA, USA). *P* values < 0.05 were considered significant.

## 3. Results

### 3.1. Lycopene Treatment Prevents U-II-Induced Cardiomyocyte Hypertrophy

**Figure 1:**
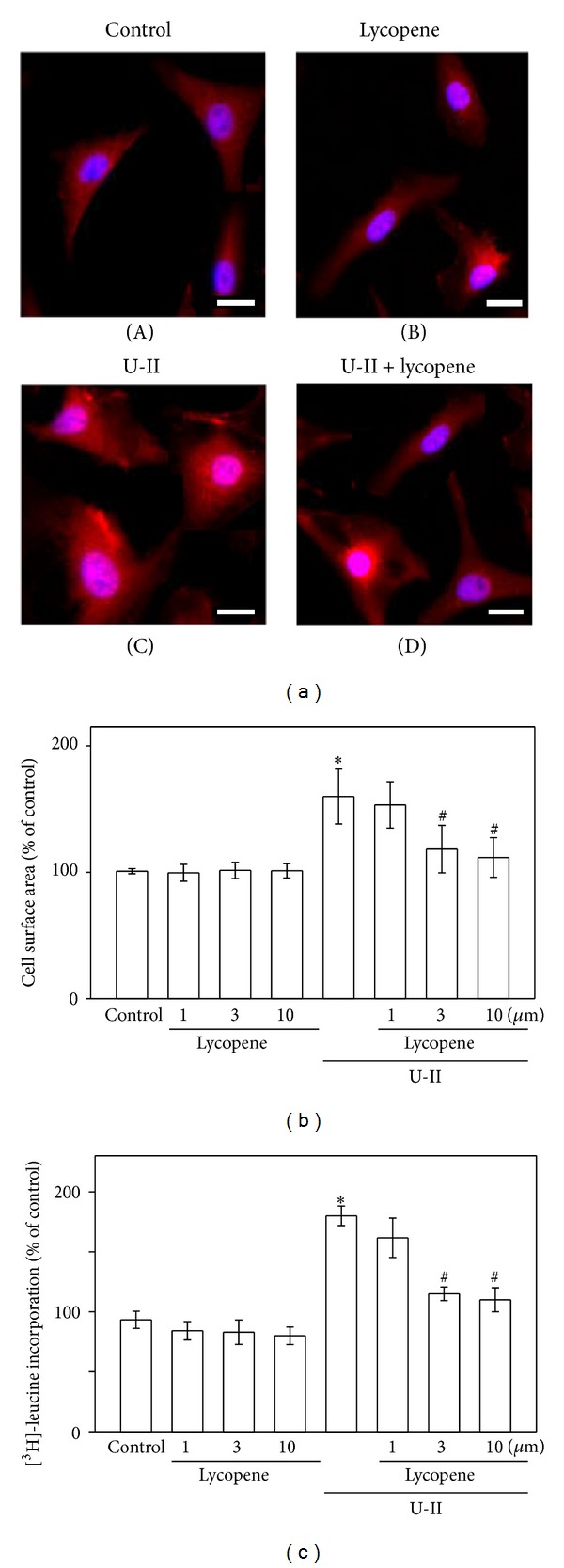
Effect of  lycopene on U-II-induced cardiomyocyte hypertrophy. (a) Effect of  lycopene on U-II-induced morphologic changes in cardiomyocytes. Cultured cardiomyocytes were exposed to vehicle control (control: top left), to lycopene alone at 3 *μ*M (lycopene: top right), or to U-II at 1 nM for 24 h in the absence (U-II: bottom left) or presence of lycopene (U-II + lycopene: bottom right) and then immunostained with an anti-*α*-actinin antibody (red), and the nucleus was stained with DAPI (blue). The figure shows a representative stain prepared based on 3 independent experiments. Scale bar shows 20 *μ*m. (b) Relative cardiomyocyte size. The cultured cardiomyocytes were exposed to vehicle control or U-II at 1 nM or 24 h in the absence or presence of lycopene (1, 3, and 10 *μ*M). The surface areas of the cardiomyocytes were measured using NIH image software in 60 randomly chosen cells from 3 dishes. The data are presented as mean ± SEM. (c) Measuring protein synthesis by using [^3^H]-leucine incorporation. The results are shown as mean ± SEM (*n* = 6). *P < 0.05 versus control; ^#^ *P* < 0.05 versus U-II alone.

Cardiomyocyte hypertrophy is characterized by an increased cell size. Therefore, we examined how lycopene affected this parameter in the U-II-treated cardiomyocytes to demonstrate the antihypertrophic effects of lycopene. The cell size significantly increased after 1 nM U-II treatment compared with that of untreated control cells (Figures 1(a) and 1(b)). Treatment with lycopene (3 and 10 *μ*M) completely ceased the increase in cell size (Figure 1(b)). Furthermore, the induction of cardiomyocyte hypertrophy is characterized by an increase in protein synthesis. U-II (1 nM) treatment enhanced the protein synthesis more compared with untreated control cells (Figure 1(c)). However, treatment with lycopene (3 and 10 *μ*M) completely ceased the increase in protein synthesis. These results suggest that lycopene prevents the development of U-II-induced cardiomyocyte hypertrophy.

### 3.2. Lycopene Inhibition of U-II-Stimulated Redox Signaling

**Figure 2:**
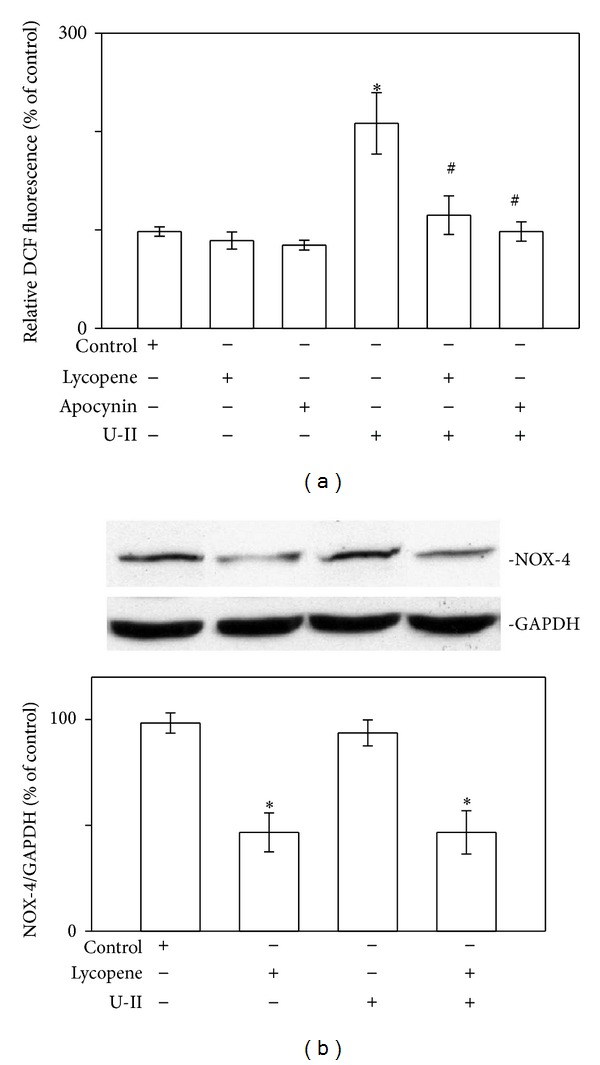
Effects of lycopene on U-II-induced ROS production in cardiomyocytes. (a) ROS levels after adding lycopene or the NAD(P)H oxidase-4 (NOX-4) inhibitor apocynin in cells exposed to U-II for 2 min. The ROS levels of cells pretreated for 6 h with lycopene (3 *μ*M) or 30 min with apocynin (0.3 mM) and exposed to U-II (1 nM) for 2 min. (b) Upper panels: representative western blot analysis of NOX-4 in cells pretreated for 6 h with lycopene (3 *μ*M) then exposed to U-II. Lower panels: the indicated values represent the ratio of NOX-4 to GAPDH. The results are shown as mean ± SEM (*n* = 6).*P < 0.05 versus control.

**Figure 3:**
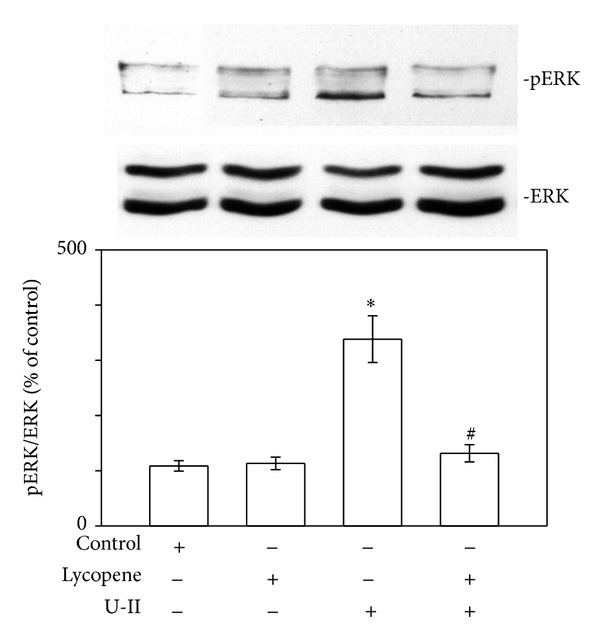
Effects of lycopene on U-II-induced ERK phosphorylation in cardiomyocytes. Upper panels: representative western blot analyses of ERK in cells pretreated for 6 h with lycopene (3 *μ*M) then exposed to U-II (1 nM) for 30 min. Lower panels: the indicated values represent the ratio of phosphorylated protein to total protein. The results are shown as mean ± SEM (*n* = 6). *P < 0.05 versus control; ^#^ *P* < 0.05 versus U-II alone.

U-II can induce ROS production, which peaked 2 min after stimulation [5], and evidence has suggested that U-II can directly activate ROS production through the induction of NAD(P)H oxidase [5, 6]. Therefore, we measured ROS levels in the absence and presence of lycopene or apocynin, an inhibitor of cellular NADPH oxidase, in cardiomyocytes exposed to U-II (Figure 2(a)). Exposing the cardiomyocytes to U-II caused increased ROS production. Pretreating the cardiomyocytes with lycopene (3 *μ*M) for 6 h or apocynin (0.3 mM) for 30 min prevented the production of U-II-induced ROS. Lycopene exerted only slight and nonsignificant inhibitory effects on ROS production in U-II-untreated cardiomyocytes. Lycopene also inhibited the increase in NOX-4 expression in cardiomyocytes (Figure 2(b)). The U-II-induced generation of ROS appears to be the primary stimulus activating ERK cascades [5]. Regarding cardiomyocytes, we investigated whether the inhibitory effect of lycopene on U-II-induced ROS production extended to the ERK signaling pathway. In our previous report, phosphorylation of ERK increased within 2 min of U-II exposure, which was sustained through 30 min of treatment [5]. Employing western blot analysis, we determined that 30 min of U-II exposure significantly increased the phosphorylation of ERK proteins in cardiomyocytes (Figure 3). Pretreatment with lycopene at a concentration of 3 *μ*M markedly reduced the U-II-induced phosphorylation of ERK proteins, suggesting that lycopene is an effective inhibitor of this pathway.

### 3.3. Lycopene Prevents GSK-3*β* Inhibition in U-II-Treated Cardiomyocytes

**Figure 4:**
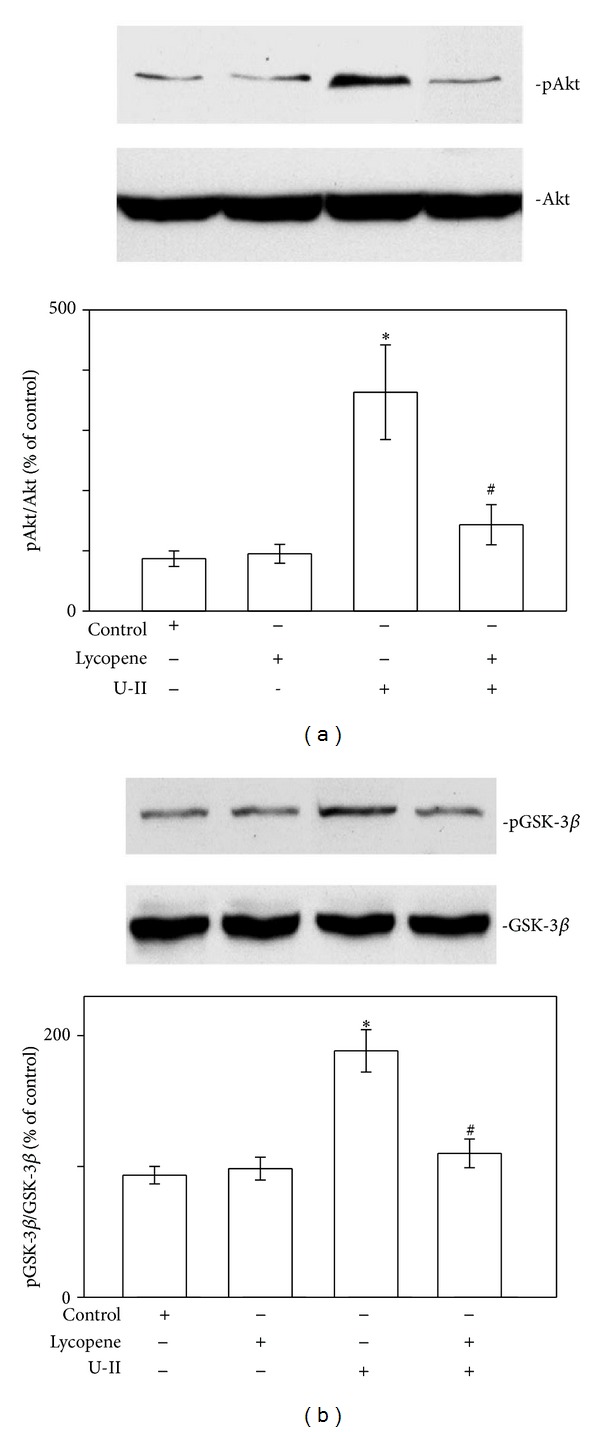
Lycopene treatment prevents Akt/GSK-3*β* phosphorylation in U-II-stimulated neonatal rat cardiomyocytes. Protein extracts from cardiomyocytes were subjected to immunoblot analysis. (a) Effects of lycopene on U-II-induced Akt phosphorylation in cardiomyocytes. Upper panels: representative immunoblots using total and antiphospho-Akt (Ser473) antibodies. Lower panels: the indicated values represent the ratio of phosphorylated protein to total protein. (b) Effects of lycopene on U-II-induced GSK-3*β* phosphorylation in cardiomyocytes. Upper panels: representative immunoblots using total and antiphospho-GSK-3*β* (Ser9) antibodies. Lower panels: the indicated values represent the ratio of phosphorylated protein to total protein. Neonatal rat cardiomyocytes were nonstimulated (control) or stimulated with U-II (1 nM for 2 min). When indicated, lycopene (3 *μ*M) was added 6 h before U-II stimulation. The results are shown as mean ± SEM (*n* = 6). *P < 0.05 versus control; ^#^ *P* < 0.05 versus U-II alone.

**Figure 5:**
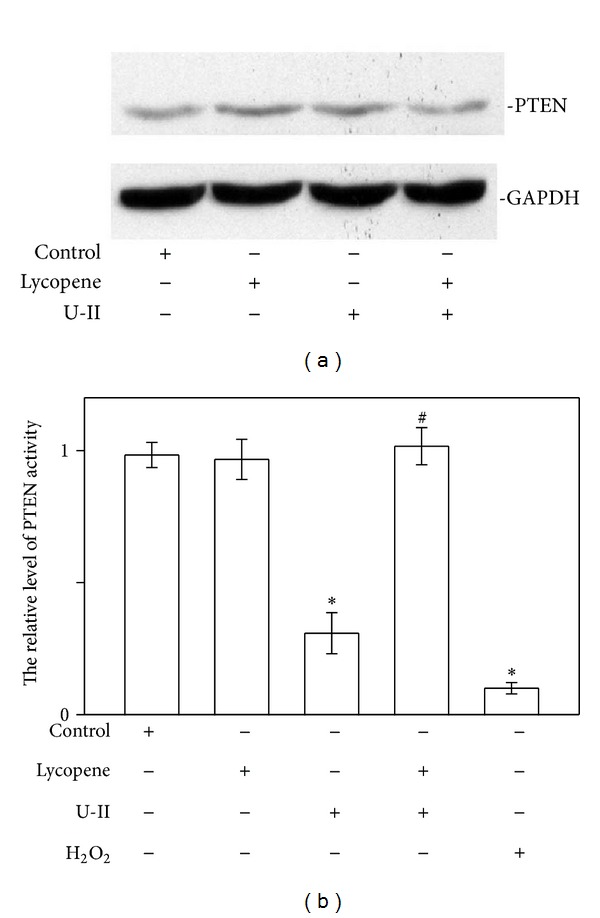
Lycopene treatment prevents PTEN oxidation in U-II-stimulated neonatal rat cardiomyocytes. (a) Lycopene does not affect PTEN protein levels in cardiomyocytes. Neonatal rat cardiomyocytes were exposed to U-II (1 nM for 1 h) in the presence or absence of lycopene (3 *μ*M) added 6 h prior to the stimuli. Representative immunoblots using anti-PTEN antibodies or anti-GAPDH antibodies are shown. The blot data are representative of 3 experiments. (b) Effect of lycopene on PTEN activity. Cardiomyocytes were treated with U-II (1 nM) in the absence or presence of lycopene (3 *μ*M) as indicated. The oxidation of the endogenous PTEN in the cardiomyocytes exposed to 0.5 mM H_2_O_2_. The level of PTEN activity in each sample was detected using a PTEN Malachite Green Assay Kit. The relative level of PTEN activity is shown as mean ± SEM (*n* = 6). *P < 0.05 versus control; ^#^ *P* < 0.05 versus U-II alone.

During the development of cardiac hypertrophy, Akt is activated and causes the phosphorylation and inactivation of the antihypertrophic kinase GSK-3*β* [19]. To test whether lycopene directly inhibited the phosphorylation of Akt and GSK-3*β*, we determined how lycopene affected the Akt/GSK-3*β* pathway during U-II-induced cardiomyocyte hypertrophy. Simulating neonatal rat cardiomyocytes with 1 nM U-II for 1 h enhanced Akt and GSK-3*β* phosphorylation (Figures 4(a) and 4(b)). Pretreatment with lycopene (3 *μ*M) markedly reduced the U-II-induced phosphorylation of the Akt and GSK-3*β* proteins. These findings demonstrate that lycopene attenuates the Akt/GSK-3*β* pathway in rat neonatal cardiomyocytes exposed to U-II. PTEN is a phosphatase that inhibits Akt signaling pathways. To elucidate the mechanism by which lycopene inhibits the activation of Akt/GSK-3*β* we evaluated whether this agent induced PTEN expression in rat neonatal cardiomyocytes. Six hours of lycopene treatment did not enhance the PTEN protein levels in neonatal rat cardiomyocytes stimulated with U-II (Figure 5(a)). Thus, we evaluated whether lycopene prevented PTEN oxidation and, consequently, its inactivation, because ROS generation inactivates PTEN [20, 21]. To determine whether lycopene prevented U-II-induced PTEN inactivation, we also investigated how lycopene affected the level of PTEN protein activity. As shown in Figure 5(b), cells exposed to U-II demonstrated decreased PTEN activity levels; by contrast, coincubating the cells with lycopene prevented the oxidation of this protein, yielding PTEN activity levels similar to those observed in control cells. Cells treated for 10 min with 0.5 mM H_2_O_2_ exhibited inhibited PTEN activity levels.

## 4. Discussion

In this study, we demonstrated that lycopene inhibits U-II-induced cardiomyocyte hypertrophy. Furthermore, the findings suggest that lycopene treatment inhibits cardiomyocyte hypertrophy through a mechanism involving the inhibition of the Akt/GSK-3*β* pathway and reduced PTEN oxidation. PTEN is a phosphatase that acts as a negative regulator of the Akt/GSK-3*β* pathway and plays a vital role in cardiac hypertrophy [22].

Similar to a previous study reporting that lycopene prevents cardiotoxicity through an antioxidant mechanism [23], we demonstrated that lycopene treatment prevented U-II-induced ROS generation. Evidence has emerged that lycopene and antioxidants exert cardioprotective effects [23–25]; however, the mechanisms of these effects remain ambiguous. In the current study, lycopene inhibited both U-II-induced ROS production and NOX-4 expression. This behavior was unsurprising because lycopene was previously reported to act as an intracellular redox agent in modulating intracellular ROS [26]. Because lycopene exhibits an extended system of conjugated double bonds, it can effectively quench the ROS generated by U-II. Moreover, recent data have suggested that lycopene inhibits NOX-4 expression in human macrophages exposed to oxidants [27, 28]. ERK have been reported to be activated by oxidant stimuli and to mediate cardiomyocyte hypertrophy [5, 17, 29]. We investigated whether the inhibitory effects of lycopene on U-II-induced cardiomyocyte hypertrophy extended to the ERK signaling pathway. Employing western blot analysis, we determined that exposing cardiomyocytes to U-II significantly increased the phosphorylation of ERK proteins; however, the phosphorylation was inhibited by lycopene, suggesting that lycopene is an effective inhibitor of ERK pathway in cardiomyocytes.

This paper is the first to suggest preventing PTEN oxidation as a mechanism for preventing ROS generation, which might contribute to the antihypertrophic effects of lycopene. Most hypertrophic stimuli cause ROS generation and PTEN is a critical target of these endogenously generated ROS [30, 31]. PTEN must be inactivated to increase cellular levels of PIP3 for recruiting downstream signaling molecules, such as Akt, and inhibiting GSK-3*β*, whose inactivation plays a vital role in the development of cardiac hypertrophy [22, 32, 33]. We demonstrated that lycopene inhibits the U-II-induced phosphorylation of Akt and GSK-3*β* by reducing ROS generation and prevents PTEN oxidation, thereby reducing its activation. These findings indicate that preventing PTEN inactivation by ROS might be a mechanism contributing to the antihypertrophic effects of lycopene. In summary, the findings suggest that lycopene prevents the U-II-induced phosphorylation of Akt/GSK-3*β* by modulating PTEN activation. These mechanisms might contribute to the antihypertrophic effects of lycopene.
